# Post-COVID Syndrome in Patients With Comorbid Hypertension or Diabetes: A Narrative Review of Long-Term Outcomes

**DOI:** 10.7759/cureus.102117

**Published:** 2026-01-22

**Authors:** Gevorg Manoukian, Sarah Kundukulam, Galeh Asatorian, Donna M Johnson, Mohd Hamza Masood, Adwaith Venugopal, Manouk Manoukian, Shruthi Aswathappa

**Affiliations:** 1 School of Medicine, St. George’s University School of Medicine, St. George’s, GRD; 2 Department of Acute Medicine and General Medicine, East Kent Hospitals NHS Trust, Kent, GBR; 3 College of Osteopathic Medicine, California Health Sciences University, Clovis, USA; 4 Department of Cardiology, Buckinghamshire NHS Trust, Aylesbury, GBR; 5 Department of Internal Medicine, International Higher School of Medicine, Bishkek, KGZ; 6 Department of Medicine, University of Central Lancashire, Preston, GBR; 7 School of Medicine, University of Colorado Anschutz Medical Campus, Aurora, USA; 8 Department of Medicine and Surgery, M.S. Ramaiah Medical College, Bengaluru, IND

**Keywords:** cardiometabolic complications, chronic disease management, covid-19 sequelae, diabetes mellitus, endothelial dysfunction, hypertension, immune dysregulation, inflammation, long-term outcomes, post-covid syndrome

## Abstract

Post-COVID syndrome (PCS), or long COVID, refers to a cluster of enduring symptoms that extend beyond the acute phase of the initial SARS-CoV-2 infection. Acute infection predominantly impacts the respiratory tract, but there is growing evidence for the multisystem involvement, such as cardiovascular, metabolic, and neurological, to be responsible for the prolonged presentation in PCS. Underlying cardiometabolic vulnerability may contribute to a high degree of susceptibility in patients with comorbidities like hypertension (HTN) and diabetes mellitus (DM). This narrative review summarizes current literature regarding PCS in patients with HTN and/or DM, focusing on proposed pathophysiological mechanisms, clinical manifestations, and reported long-term outcomes. In these populations, PCS has been linked across studies to processes including endothelial dysfunction, chronic low-grade inflammation, autonomic imbalance, and potential dysregulation of the renin-angiotensin-aldosterone system (RAAS). Persistent cardiovascular, metabolic, and neurocognitive symptoms are reported, but the magnitude and patterns of risk vary across studies, while comparative findings across HTN and DM remain heterogeneous. Symptoms reported frequently include fatigue, cognitive impairment (“brain fog”), and psychological distress, supporting the multisystem complexity of PCS. Although, previous work has indicated that cardiometabolic comorbidities could interact and moderate PCS severity and persistence, there is an important shortfall of both causality and prognosis, as well as the management of PCS. Longitudinal studies are needed for future research regarding risk stratification, disease course, and targeted interventions in individuals with PCS with comorbid high blood pressure and diabetes.

## Introduction and background

Post-COVID syndrome (PCS), also known as long COVID, is characterized by a spectrum of enduring illnesses and organ dysfunction beyond four to 12 weeks of an initial acute SARS-CoV-2 infection [1]. The World Health Organization has identified PCS as a multisystem disease that presents with symptoms such as fatigue, shortness of breath, and cognitive dysfunction in addition to various cardiometabolic dysfunctions that contribute to morbidity and the burden of healthcare [2]. Estimates indicate that 10% to 30% of COVID-19 survivors form long-term sequelae, and thus make it imperative to study recovery trajectories in vulnerable populations [3].

Hypertension (HTN) and diabetes mellitus (DM) are consistently cited comorbidity risk factors correlated with higher PCS risk and longer duration of symptomatology [4]. Key contributors to this association are endothelial dysfunction, chronic low-grade inflammation, and immune dysregulation, common to PCS and cardiometabolic disease. Acute SARS-CoV-2 infection may amplify these existing dysfunctions and result in a destabilization of blood pressure and glycemic control in vulnerable subjects [5]. Other mechanisms, including angiotensin-converting enzyme 2 (ACE2)-mediated vascular inflammation, cytokine-driven metabolic compromise, and pancreatic involvement, have also been suggested but are not fully defined in PCS [6,7]. These pathways should thus be seen as biologically plausible hypotheses instead of fully established drivers of disease.

Although PCS has gained more attention in the setting of HTN and DM, there are still significant gaps around the relative impact of these several mechanisms, their longitudinal evolution, and the implications for prognosis and management. In this current narrative review, we have sought to comprehensively summarize the available evidence regarding PCS in individuals with pre-existing HTN and/or DM, specifically focusing on intertwined mechanisms contributing to their prolonged and frequently severe clinical courses. We aim to investigate these common vascular, metabolic, and immunologic pathways that render this population prone to long-term sequelae, as well as underscore the lack of evidence surrounding their most appropriate follow-up and management. Through integration and by identifying gaps in the literature, our review aims to offer clinicians and researchers a clear framework for recognizing, monitoring, and managing the long-term impact of PCS on this vulnerable population.

## Review

Methods

This narrative review was conducted to examine the literature on the long-term effects of PCS among patients with a history of HTN and/or DM. An extensive search of PubMed, Scopus, and Google Scholar was conducted for articles published from January 2020 to September 2025, together with a manual reference screening of full-text articles to identify additional relevant studies. The search strategy combined controlled vocabulary and free text terms related to “Post-COVID syndrome,” “Long COVID”, “SARS-CoV-2 sequelae”, “hypertension”, “ diabetes mellitus”, “pathophysiology”, “endothelial dysfunction”, “RAAS dysregulation”, and “long-term outcomes”. The eligible literature consisted of peer-reviewed original research articles, systematic reviews, and meta-analyses published in English that reported post-acute (>4-12 weeks) or long-term sequelae of COVID-19 in patients with pre-existing hypertension or diabetes mellitus. A manuscript was considered ineligible if it focused exclusively on acute COVID-19, did not provide subgroup analysis for these comorbidities, or was a non-peer-reviewed commentary, preprint, or anecdotal case report. No exclusions were applied based on the severity of COVID-19, which ranged from mild to severe across included studies. Screening and data extraction were independently conducted by multiple reviewers, and this included study design, sample size, comorbid profiles, timeframe of follow-up, and primary cardiovascular, renal, metabolic, and neuropsychiatric outcomes. Findings were synthesized narratively and organized thematically to address common mechanisms, late sequelae, relative risks, and multidisciplinary management. Greater interpretive weight was given to high-quality studies published in peer-reviewed journals (such as Nature Medicine, The Lancet, British Medical Journal (BMJ), and JAMA Cardiology). No formal bias scoring was performed due to the narrative nature; however, critical appraisal focused on clear methodology, reproducibility, and relevance to the PCS population. A small number of pre-2020 mechanistic studies were included only when essential in describing underlying vascular, inflammatory, or metabolic pathways relevant to post-COVID pathophysiology and are distinct from modern clinical data to maintain the quality of the evidence base.

Discussion

This discussion synthesizes current evidence on the relationship between PCS, HTN, and DM, focusing on shared pathophysiological pathways, long-term multisystem outcomes, and implications for clinical management. Rather than detailing individual studies in isolation, the following sections integrate findings across cardiovascular, metabolic, renal, neurological, and autonomic domains to highlight convergent mechanisms and clinically relevant patterns observed in high-risk populations.

Pathophysiological Links Between PCS, HTN, and DM

Traditionally, HTN and DM have been well studied as separate entities and for their effects on each other. Since the onset of COVID-19, clear pathophysiological links between them and PCS have been found. In this section, we understand these links by which PCS can affect patients with long-standing HTN and DM. The endothelium is a single cell layer inside blood vessels that controls the transportation of materials into and out of the blood, regulates inflammation and immunity, senses mechanical stresses from blood flow, and releases signaling molecules that influence blood pressure and vessel health [8]. This process has been associated with a hypercoagulable state and may contribute to microvascular injury [9]. This mechanism is important to understand for patients with co-existing HTN, as research has shown that endothelial damage and HTN reinforce each other [10]. PCS has been associated with endothelial dysfunction, which may partially explain reports of new-onset hypertension and worsening blood pressure control in susceptible individuals, which become difficult to manage due to vascular stiffening secondary to endothelial damage [11].

HTN and DM are chronic illnesses that cause the body to remain in a hyperinflammatory state, leading to increased levels of C-reactive protein (CRP), interleukin-6 (IL-6), and tumor necrosis factor alpha (TNF-α) [12]. Tests done in patients with PCS have shown raised levels of CRP and fibrinogen, which have been studied as sequelae of IL-6 production during COVID-19 infection [13]. Consistently raised inflammatory markers may lead to increased metabolic and vascular stress, thereby potentially leading to endothelial dysfunction and increased insulin resistance [14]. Dysautonomia is an umbrella term that describes a disorder of the autonomic nervous system (ANS). The ANS controls bodily functions such as the heart rate, blood pressure, temperature, digestion, and breathing. The most common symptom of dysautonomia is postural hypotension/dizziness. PCS in patients with ongoing problems with HTN and DM are at high risk for dysautonomia, which can be further exacerbated by PCS [15]. Studies suggest postural orthostatic tachycardia syndrome and orthostatic hypotension are caused by autoantibodies to α-/β-adrenoceptors and muscarinic receptors, leading to vasodilation. It has been hypothesized that SARS-CoV-2 infection may trigger immune-mediated autonomic responses [16]. Another hypothesis suggests that PCS may contribute to dysautonomia by inducing sympathetic activation, which in turn triggers the release of pro-inflammatory cytokines. This leads to overstimulation of the vagus as an anti-inflammatory response [17]. This vagal response initiates the “inflammatory reflex”, in which afferent vagal signals detect elevated levels of inflammatory mediators and trigger an efferent parasympathetic (vagal) response that diminishes further inflammatory damage. Here, the efferent vagus nerve activation causes the release of acetylcholine, which binds to α7 nicotinic acetylcholine receptors on macrophages, which suppresses the production of pro-inflammatory cytokines such as TNF-α and IL-6 [18-20]. This mechanism may represent one pathway through which autonomic regulation attempts to restore inflammatory and vascular balance following SARS-CoV-2 infection: the initial sympathetic and cytokine surge is partly counter-regulated by vagal (parasympathetic) output, which limits excessive inflammation and associated tissue and vascular damage [21,22].

**Figure 1:**
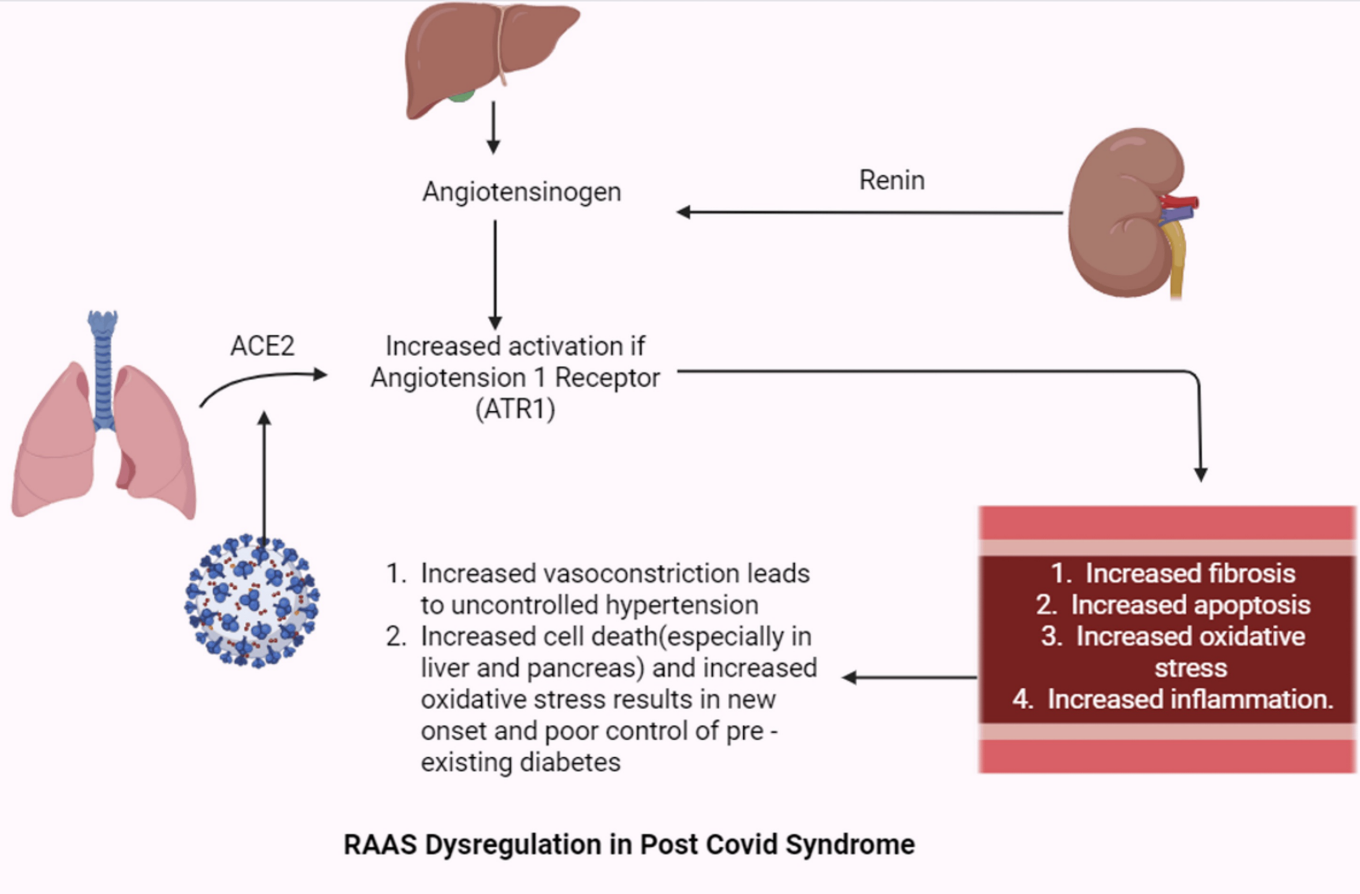
Schematic representation of the RAAS dysregulation during SARS-CoV-2 infection. Data adapted from [24,25]. ACE2: angiotensin-converting enzyme 2; RAAS: renin-angiotensin-aldosterone system Image credits: Mohd Hamza Masood; Figure created in Biorender.com: https://app.biorender.com/illustrations/6904bde75fb24b96b2db3382.

The renin-angiotensin-aldosterone system (RAAS) is responsible for regulating acute and chronic alterations in blood pressure by managing blood volume, electrolyte balance, and systemic vascular resistance through the maintenance of vascular tone and salt and water homeostasis [23]. Knowing this, it is clear how important RAAS is in managing HTN and how it can help protect the kidneys from DM [23,24]. RAAS dysregulation has been observed in patients with PCS. SARS-CoV-2 activates the RAAS by attaching, via its spike protein, to the ACE2, which is involved in the degradation of angiotensin II. This, in turn, leads to the overactivation of the angiotensin 1 receptor (AT1R). AT1R causes detrimental effects such as vasoconstriction, fibrosis, apoptosis, oxidative stress, and inflammation (Figure 1). The vasoconstriction results in poorly controlled HTN in patients predisposed to it. The attachment to the ACE2 receptor enables SARS-CoV-2 to travel to multiple body parts and cause a range of symptoms [25]. For diabetics, the recovery from PCS is difficult to manage, as ACE2 allows COVID to enter key organs like the liver and pancreas, causing cell death and insulin resistance. This mechanism also results in many cases of new-onset DM [25]. Treatment of COVID-19 with long-term steroids also increases the number of patients at high risk of DM to new-onset DM [25]. Furthermore, SARS-CoV-2 causes dysregulation of sodium/glucose cotransporter 1 (SGLT1) in the intestinal epithelium. Upregulation of SGLT-1 leads to increased intestinal glucose absorption, which facilitates the development of hyperglycemia in diabetic patients [25].

Long-Term Outcomes in Hypertensive Patients with PCS

**Figure 2:**
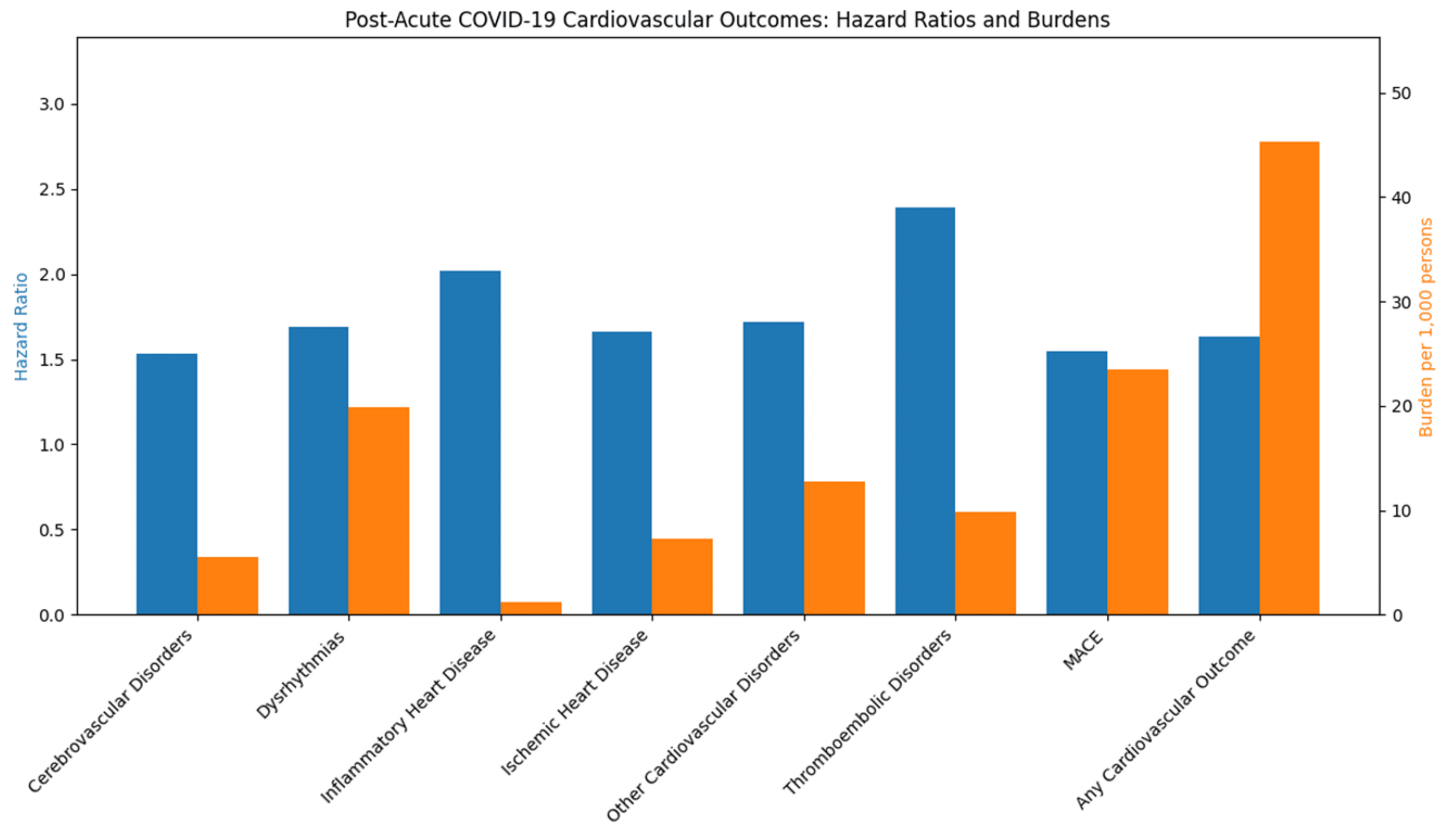
Bar graph illustrating the hazard ratios and burdens of post-acute COVID-19 cardiovascular outcomes. Data adapted from [26]. MACE: major adverse cardiovascular events Image credits: Galeh Asatorian

Across large observational cohorts and meta-analyses, PCS has been consistently associated with an elevated burden of cardiovascular sequelae, including dysrhythmias, ischemic heart disease, inflammatory cardiac conditions, and thrombotic events up to one year following acute infection (Figure 2) [26]. These associations have been observed across both hospitalized and non-hospitalized populations and appear to persist even among individuals without pre-existing hypertension, suggesting that SARS-CoV-2 infection may confer independent long-term cardiovascular risk. In patients with underlying HTN, baseline vascular dysfunction may further amplify susceptibility, although current evidence remains largely associative rather than causal.

Beyond cardiovascular involvement, PCS has been consistently associated with adverse long-term renal outcomes, particularly among individuals with pre-existing cardiometabolic comorbidities. Meta-analytic evidence indicates that a substantial proportion of patients who develop COVID-19-associated acute kidney injury experience incomplete renal recovery months after infection, with progression to chronic kidney disease or persistent dialysis dependence observed in a notable minority [27]. Even among individuals without documented acute kidney injury during the initial infection, post-COVID follow-up studies demonstrate measurable declines in estimated glomerular filtration rate, suggesting subclinical or delayed renal involvement. Importantly, comorbid conditions such as HTN, cardiovascular disease, and baseline renal impairment appear to amplify susceptibility to long-term renal dysfunction, although heterogeneity in infection severity and follow-up duration limits causal inference.

**Figure 3:**
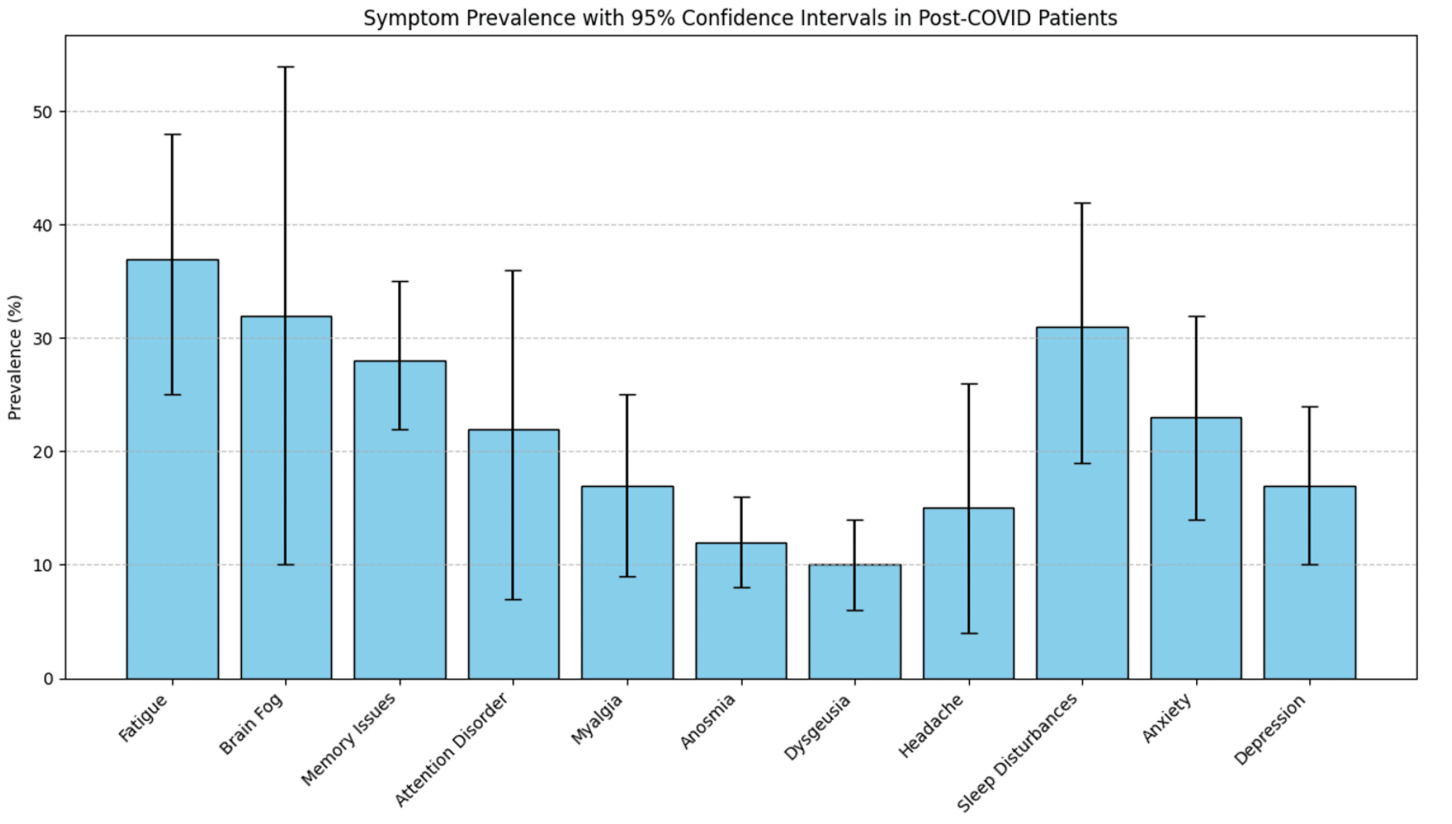
Error bar chart comparing the prevalence of different neurological and psychiatric symptoms in post-COVID patients. Data adapted from [28]. Image credits: Galeh Asatorian

PCS has also been associated with a broad spectrum of persistent neurological and neuropsychiatric manifestations extending months beyond acute infection. Pooled analyses across large patient cohorts indicate a high prevalence of fatigue, cognitive complaints (including brain fog, memory impairment, and attentional difficulties), myalgia, and headache, alongside neuropsychiatric symptoms such as sleep disturbances, anxiety, and depression (Figure 3) [28]. These symptoms have been observed across both mid-term and long-term follow-up periods, with several, including anxiety and depressive symptoms, showing persistence or increasing prevalence over time. Collectively, these findings suggest that neurological and neuropsychiatric sequelae represent a substantial and enduring component of PCS, with potential implications for functional capacity and quality of life, particularly in patients with underlying cardiometabolic disease.

Long-Term Outcomes in Diabetic Patients with PCS

**Figure 4:**
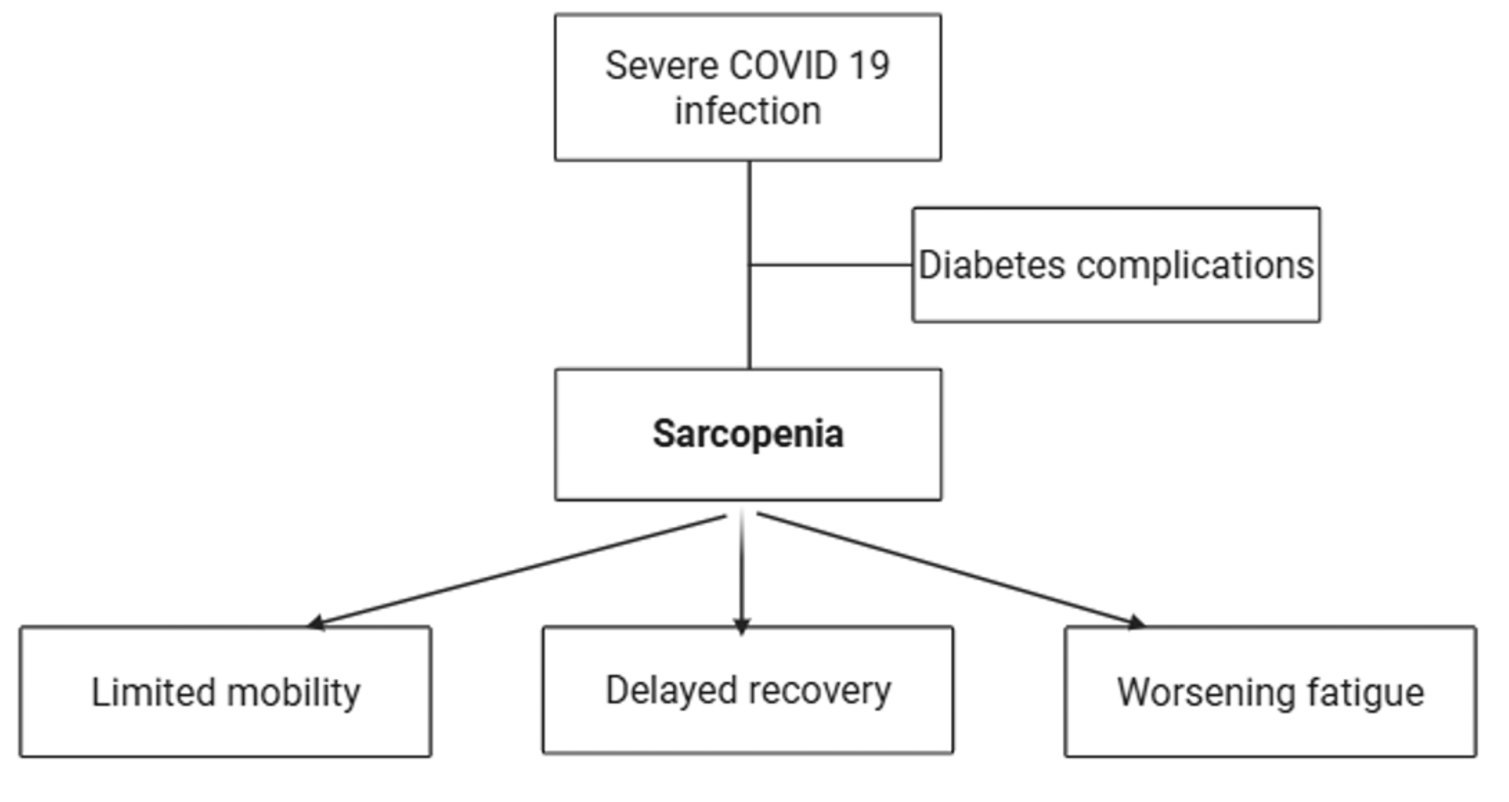
Flow chart showing effects of post-COVID syndrome in people with diabetic complications. Data adapted from [33]. Image credits: Donna Johnson

Poor glycemic control appears to have a bidirectional relationship with PCS. Studies suggest that poorer baseline glycemic control is associated with more severe PCS manifestations and adverse outcomes, while COVID-19 itself may contribute to worsening metabolic control [29]. Similarly, it was noted that COVID-19 itself has led to progression of DM in terms of worsening control or progressing from pre-diabetes to DM, and a significant number of steroid-induced diabetes cases have been identified [30]. COVID-19 has also been temporally associated with new-onset autoimmune diabetes in genetically predisposed individuals, although causality has not been definitively established [31]. A multicenter study done in London during the pandemic also reported an increased number of type 1 DM diagnoses and diabetic ketoacidosis cases in children up to 16 years of age [32]. Fatigue and physical deconditioning in diabetic PCS patients appear to reflect the combined effects of post-viral inflammation, diabetes-related neuropathy and myopathy, prolonged inactivity, and corticosteroid exposure, collectively increasing the risk of sarcopenia and delayed functional recovery (Figure 4) [33].

It is well established that DM can lead to complications in the eyes, nerves, and kidneys by damaging tiny blood vessels. However, what remains uncertain is whether the microvascular damage caused by a COVID-19 infection could worsen these pre-existing conditions. Interestingly, there are theories that damage to capillaries, including cell swelling and scarring, may be the root cause of many post-COVID symptoms like fatigue, 'brain fog', and memory problems. The most alarming possibility for diabetic patients is that such extensive capillary damage could potentially trigger severe organ failure, particularly in the kidneys. However, this area needs further research to further evaluate the impact with the help of statistics [30]. Postural tachycardia syndrome (POTS), characterized by an orthostatic increase in heart rate of over 30 beats per minute within 10 minutes of standing, presents another challenge [34]. The underlying pathophysiology and long-term implications of this condition remain to be fully elucidated. In patients with long-standing DM, pre-existing autonomic neuropathy may already predispose them to tachycardia and postural hypotension. The co-existence of POTS can therefore exacerbate these symptoms, leading to heightened fatigue, giddiness, and tachycardia, and contributing to the overall clinical presentation of PCS [30].

COVID-19 is known to cause immunosuppression, thus increasing the risk of secondary infections. There are multiple mechanisms for this, the most common one being an increase in pro-inflammatory markers leading to cytokine storm and a decrease in CD4+ and CD8+ T cells and B cells. Over-cytotoxicity of CD8+T cells can also lead to overactivation of T cells, which can further lead to immunosuppression [35]. Airway tissue and blood vessel damage caused by COVID also increases the risk of co-infections [36]. Another mechanism seen is the increased affinity of SARS-CoV to ACE2 receptors present in the oral mucosa, thus increasing the risk of oral infections [37]. Looking at a case report from India, there seems to be an increase in fungal infections like mucormycosis and candidiasis, and bacterial infections like actinomycosis in diabetic patients. The main predisposing factors for this were concluded to be poor glycemic control and prolonged steroid treatment for COVID [38]. A retrospective observational study done in Sweden over a two-year period with a population group consisting of people with PCS symptoms showed a higher rate of common bacterial infections amongst diabetic people. The study also showed that being overweight was an additional risk factor for female patients with diabetes [39]. It's been well-documented that COVID-19 can cause severe heart inflammation and even heart failure, particularly in hospitalized patients. While other viruses can cause similar lingering effects, our findings show that even healthy individuals with PCS can have subtle heart issues. What we don't know is the long-term significance of these findings or if they will lead to lasting damage like scarring of the heart muscle. The role of pre-existing heart problems in the development of PCS also needs to be explored. Furthermore, another finding worth investigating is the connection between pancreatic damage and the higher rate of new DM in PCS patients [40].

Comparative Risk: HTN vs DM

A descriptive study was done in Turkey, including healthcare professionals who tested positive for COVID-19 four weeks prior, to investigate further PCS and its relation to their comorbidities and lifestyle factors. A variety of symptoms were identified, with forgetfulness, fatigue, and weakness being the most common ones; 3.6% of the chosen study group were diabetic, and the most common PCS symptoms they encountered were forgetfulness, cough, irritability, and numbness in hands and legs. Similarly, 3.6% of the chosen population was hypertensive, and the most common PCS symptoms they experienced were muscle pain, forgetfulness, dizziness, vision problems, a feeling of fullness in the ear, loss of concentration, and diarrhea. This study has two key limitations: its small scale and an ambiguous methodology that does not clarify if the reported comorbidities were found together in the same individuals or in separate participants [41]. Another cohort study following up patients six and 12 months post-discharge from hospitalization with COVID-19 showed that PCS was notably more prevalent in patients with high blood pressure and diabetes six months after their hospitalization. The prevalence rates were 98.2% and 98.4%, respectively, which were significantly higher than the rates in individuals without these comorbidities. However, by 12 months, while the prevalence of the syndrome in the high blood pressure group (81.6%) remained significantly higher than in the control group, the difference for the diabetes group (77.1%) was no longer statistically significant. This suggests that while both conditions increase the short-term risk of PCS, the long-term impact may differ [42]. DM is the most significant pre-existing cardiovascular risk factor for the development of post-acute sequelae of COVID-19 (PASC), elevating risk by a factor of 4.39. This finding is consistent with existing evidence that links diabetes to more severe COVID-19 progression. The proposed mechanism for post-COVID symptoms involves endothelial damage, heightened oxidative stress, and increased pro-inflammatory cytokines. These physiological processes can increase the risk of thromboembolism, pulmonary fibrosis, and acute respiratory distress syndrome, thereby contributing to more severe acute illness and a higher probability of long-term symptoms [43]. The study found that HTN is the third most significant risk factor for developing long COVID in older adults, tripling their likelihood of developing the condition. The systemic inflammatory response to COVID-19 can alter blood vessel cells, potentially causing long-term kidney and vascular dysfunction. This could explain how HTN intensifies the chronic inflammation in acute COVID-19 patients, thereby contributing to long-term symptoms [43]. This conclusion is supported by other research, including a case-control study that found a link between pre-existing HTN and a greater number of long-term COVID symptoms [44].

Management Strategies and Follow-up Recommendations

Effective management of PCS in patients with HTN or DM requires coordinated multidisciplinary follow-up. Cardiovascular sequelae, including myocarditis, arrhythmias, and autonomic dysfunction, remain prevalent. Structured cardiology input, such as echocardiography and ambulatory monitoring, is therefore recommended within the first three to six months post infection [45,46]. A study demonstrated that cardiac involvement is present in 78% of patients recovering from COVID-19, with ongoing myocardial inflammation in 60% of cases, regardless of pre-existing conditions or the severity of the acute illness [46]. These findings underscore the need for continuous cardiology monitoring in the post-acute phase to assess long-term cardiovascular consequences of COVID-19. Endocrinology oversight is also crucial for detecting post-viral dysglycemia, steroid-induced hyperglycemia, and fluctuating insulin sensitivity, all of which complicate diabetes control [47]. Moreover, nephrological surveillance is essential due to the risk of persistent proteinuria, reduced estimated glomerular filtration rate (eGFR), and accelerated chronic kidney disease progression in this population [48]. Rehabilitation specialists play a pivotal role in assessing exercise tolerance, prescribing graded reconditioning programs to improve physical strength and endurance, and mitigating deconditioning or sarcopenia [49]. Integrated multidisciplinary team clinics, whether virtual or in-person, facilitate synchronized risk stratification, shared care planning, and early detection of organ-specific complications, thereby improving long-term outcomes in high-risk individuals [50]. A study on multidisciplinary rehabilitation programs showed significant reductions in hospital readmissions for patients with long COVID. The experimental rehabilitation program reduced the relative risk of hospital readmissions by 85.7%, while other rehabilitation strategies reduced this risk by 42.0% and 66.0%, respectively. These findings suggest that a tailored, multidisciplinary rehabilitation program not only provides short-term benefits but also long-term preventive effects, including reducing the onset of disabilities, medication use, and the need for specialist consultations over the next six months. The study calls for future research to identify the most effective and cost-efficient rehabilitation strategies for patients with long COVID [51]. These results align with emerging post-COVID care frameworks from the UK's National Health Service (NHS) recommendations [45].

The question of whether more intensive cardiometabolic control improves PCS outcomes remains unresolved. Current evidence does not support treatment targets lower than established HTN or DM guidelines. Standard recommendations for blood pressure <130/80 mmHg in most high-risk adults and individualized HbA1c goals (generally <7% for many non-pregnant adults) remain appropriate [52,53]. Recent research highlights a heightened incidence of new-onset HTN and DM among COVID-19 survivors (Table 1). A large-scale review reported that SARS-CoV-2 infection was associated with a 1.4-1.8-fold increase in post-COVID HTN [54]. Proposed mechanisms include endothelial dysfunction, persistent inflammation, dysregulation of the RAAS, and autonomic imbalance, all contributing to long-term vascular and blood pressure abnormalities. These findings support proactive cardiovascular follow-up and closer monitoring in high-risk individuals, rather than deviation from established treatment targets. Regarding glycemic status, one study found an incidence of 15.8 per 1000 person-years of new-onset type 2 DM in COVID-19 survivors compared with 12.3 per 1000 person-years in matched controls, with an incidence rate ratio of 1.28 (95% CI 1.05-1.57) [55]. Another study further observed that long COVID often presents with autonomic dysregulation, vascular stiffness, and renal microvascular injury, exacerbating metabolic instability in patients with DM [56]. Routine screening for fasting glucose and HbA1c at three to six months post infection is therefore recommended in high-risk individuals. Evidence for “aggressive” control remains lacking; overly stringent targets may increase risks of hypoglycemia, hypotension, and treatment burden. Guideline-directed optimization with enhanced monitoring remains the most evidence-based approach for PCS management.

**Table 1:** Summary of key studies demonstrating increased cardiometabolic risk following COVID-19 infection. SARS-CoV-2 infection has been linked to higher rates of new-onset HTN and DM, as well as autonomic and metabolic dysregulation among long COVID patients. HTN: hypertension; DM: diabetes mellitus; RR: relative risk; IRR: incidence rate ratio; AURI: acute upper respiratory infection (control group).

Study	Outcome	Population	Incidence/Effect Size	Comparator	Reported Risk/Findings
Teymourzadeh et al., 2025 [54]	New-onset HTN	Post-COVID adults (systematic review and meta-analysis)	-	Non-COVID controls	SARS-CoV-2 infection associated with 1.4–1.8 fold higher risk of post-COVID HTN
Rathmann et al., 2022 [55]	New-onset type 2 DM	COVID-19 survivors (n=35,865)	15.8 vs 12.3 per 1000 person-years	Matched AURI controls	IRR 1.28 (95% Cl 1.05–1.57) for new onset DM
Matsumoto et al., 202s [56]	Autonomic & metabolic dysregulation	Long COVID patients with DM	–	–	Long COVID associated with autonomic imbalance, vascular stiffness, and renal microvascular injury, worsening glycemic control

In PCS, particularly among individuals with comorbidities such as HTN or DM, structured lifestyle and rehabilitation interventions are pivotal to recovery. Meta-analytic evidence underscores this need; among 4,828 PCS patients across 12 studies, 59% (95% CI: 42-75%) reported poor quality of life on the EuroQol Visual Analogue Scale (EQ-VAS) [57]. Quantitative studies further support the effectiveness of multidisciplinary rehabilitation; a six-week outpatient program achieved a mean improvement of 112 meters in walking distance and significant reductions in fatigue and breathlessness [58]. Consistent findings across additional clinical cohorts demonstrate improvements in functional independence, pulmonary capacity, and overall quality of life following combined physical, respiratory, and psychological rehabilitation strategies [59,60]. Collectively, these data establish rehabilitation as a cornerstone of evidence-based PCS management. Exercise prescriptions should be graduated and individualized, with pacing strategies to avoid post-exertional symptom exacerbation. The American Heart Association emphasizes that early rehabilitation must prioritize short-duration, low-intensity exercise to reduce the risk of post-exertional malaise. Upright modalities such as walking or running may worsen orthostatic intolerance; semi-recumbent exercises such as cycling or rowing are recommended initially, followed by gradual progression [61]. This aligns with National Institute for Health and Care Excellence (NICE) NG188, which highlights pacing, individualized goal-setting, and holistic rehabilitation within multidisciplinary care models [45]. Nutritionally, whole-food dietary patterns, particularly the Mediterranean diet, show emerging benefits in long COVID. The BioICOPER study (2024) found that higher adherence to a Mediterranean diet in long COVID cohorts was associated with fewer metabolic syndrome components, smaller waist circumference, and higher high-density lipoprotein (HDL)-cholesterol [62]. Similarly, a pilot trial of individualized nutrition therapy combined with tailored physical rehabilitation reported enhanced functional performance versus standard physiotherapy [63]. Emerging approaches such as caloric restriction or fasting are under investigation, signaling potential for focused dietary interventions. Given the interplay of post-viral inflammation, metabolic disruption, and deconditioning in hypertensive or diabetic patients, a Mediterranean-style diet emphasizing fruits, vegetables, whole grains, lean proteins, and unsaturated fats is a reasonable first line, with supplementation reserved for documented deficiencies.

Psychological support is vital for high-risk PCS patients, especially those with comorbidities like HTN or DM. Depression, anxiety, and post-traumatic stress disorder (PTSD) are common, compounding physical health burdens [64]. Cognitive-behavioral therapy and mindfulness-based stress reduction effectively reduce anxiety and depression in post-COVID populations [65]. Trauma-focused interventions such as eye movement desensitization and reprocessing (EMDR) or prolonged exposure therapy help manage PTSD [65]. Integrating psychological care into multidisciplinary models fosters resilience and improves health outcomes [66]. Telehealth has also expanded access to essential mental health services during the pandemic, particularly for underserved populations [67].

Gaps in Knowledge and Future Research Directions

Despite the above theories highlighting a potential association between certain comorbidities and the prognosis of PCS, they reveal significant knowledge gaps that affect the understanding of the exact mechanisms behind this association and, subsequently, the optimal management strategies to prevent PCS complications.

Firstly, a major challenge faced is that most of the research conducted on this topic includes longitudinal studies, which either lack a sufficient follow-up period to identify long-term prognoses or the validity of obtained data is constrained by small sample sizes [68]. Secondly, many studies utilize self-reported clinical features provided by patients, and there are often inconsistencies in the definition of PCS, as well as a lack of quantifiable data, which leads to poor validity of the evidence obtained [69]. Finally, studies have discussed that from a pathophysiology point of view, the exact mechanisms of how diabetic and hypertensive control influence the risk of post-COVID complications are still poorly understood, despite a few studies linking the co-morbidities to the potential for immunosuppression, chronic inflammation, and endothelial injury, which may influence PCS prognosis [70, 71].

Considering the above findings, future research should aim to establish an objective and well-defined criterion for PCS, based on functional assessments, clinical and biochemical features, to improve the standardization of study results. They should also prioritize large-scale, longitudinal cohort studies with longer-term follow-ups for high-risk patients impacted by COVID-19. Moreover, research should investigate further into the direct impact of strict blood pressure and glycemic level control in diabetic and/or hypertensive patients affected by COVID-19 on their risk of developing severe complications of PCS. Studies investigating the role of COVID-19 vaccinations in preventing the incidence of PCS in these high-risk patients have suggested the need for observational studies to accurately examine their role in reducing the risk and duration of long-haul COVID [72]. Addressing these concerns could provide further clarity on how PCS-related organ damage interacts with poorly controlled HTN and DM, as well as formulate comprehensive public health strategies against long-haul COVID.

## Conclusions

This narrative review synthesizes current evidence indicating that individuals with HTN or DM represent a high-risk population for post-COVID syndrome, with increased susceptibility to persistent cardiovascular, metabolic, renal, neurological, and autonomic sequelae. Across diverse study designs, PCS appears to reflect the convergence of endothelial dysfunction, chronic inflammation, autonomic imbalance, and metabolic stress mechanisms that may be amplified in patients with pre-existing cardiometabolic disease. While COVID-19 and its therapies are associated with worsened blood pressure and glycemic control, current evidence does not establish that stricter-than-standard cardiometabolic targets independently modify PCS outcomes. Instead, available data support guideline-directed management combined with proactive surveillance, multidisciplinary follow-up, and individualized rehabilitation strategies to mitigate long-term morbidity. Clinically, these findings underscore the importance of early identification of high-risk individuals, structured post-acute monitoring, and integrated care pathways addressing cardiometabolic, functional, and psychological recovery. Future large-scale longitudinal studies with standardized PCS definitions are required to clarify causal mechanisms, identify prognostic markers, and determine whether targeted interventions can meaningfully alter long-term outcomes in vulnerable populations.
